# HBpF-proBDNF: A New Tool for the Analysis of Pro-Brain Derived Neurotrophic Factor Receptor Signaling and Cell Biology

**DOI:** 10.1371/journal.pone.0150601

**Published:** 2016-03-07

**Authors:** Perrine Gaub, Andrès de Léon, Julien Gibon, Vincent Soubannier, Geneviève Dorval, Philippe Séguéla, Philip A. Barker

**Affiliations:** Montreal Neurological Institute, Department of Neurology and Neurosurgery, McGill University, Montreal, Quebec, H3A 2B4, Canada; University of Louisville, UNITED STATES

## Abstract

Neurotrophins activate intracellular signaling pathways necessary for neuronal survival, growth and apoptosis. The most abundant neurotrophin in the adult brain, brain-derived neurotrophic factor (BDNF), is first synthesized as a proBDNF precursor and recent studies have demonstrated that proBDNF can be secreted and that it functions as a ligand for a receptor complex containing p75NTR and sortilin. Activation of proBDNF receptors mediates growth cone collapse, reduces synaptic activity, and facilitates developmental apoptosis of motoneurons but the precise signaling cascades have been difficult to discern. To address this, we have engineered, expressed and purified HBpF-proBDNF, an expression construct containing a 6X-HIS tag, a biotin acceptor peptide (BAP) sequence, a PreScission^™^ Protease cleavage site and a FLAG-tag attached to the N-terminal part of murine proBDNF. Intact HBpF-proBDNF has activities indistinguishable from its wild-type counterpart and can be used to purify proBDNF signaling complexes or to monitor proBDNF endocytosis and retrograde transport. HBpF-proBDNF will be useful for characterizing proBDNF signaling complexes and for deciphering the role of proBDNF in neuronal development, synapse function and neurodegenerative disease.

## Introduction

The four mammalian neurotrophins comprise a family of related secreted factors required for differentiation, survival, development and death of specific populations of neurons and non-neuronal cells. The effects of the neurotrophins are mediated by binding to TrkA, TrkB and TrkC receptor tyrosine kinases and to the p75 neurotrophin receptor (p75NTR). The Trk receptors play critical roles mediating neuronal survival and growth and are important modulators of synaptic function [1]. p75NTR is a component of distinct cell surface signaling platforms that function to induce apoptosis and mediate neuronal growth inhibition. However, p75NTR also acts as a Trk co-receptor that increases the binding specificity and affinity of Trk receptors for neurotrophins [2, 3].

Neurotrophins are produced as proforms of approximately 240 amino acids that are cleaved by furins and proconvertases to yield mature neurotrophins of about 120 amino acids [3]. The main functions ascribed to the neurotrophin prodomain include facilitating neurotrophin folding and directing neurotrophins to the regulated secretory pathway [4–6]. Several recent studies have indicated that nerve growth factor (NGF) and brain-derived neurotrophic factor (BDNF) are secreted from cells as prodomain-containing forms (proNGF and proBDNF, respectively) [7–9]. In some types of primary neurons and in endothelial cells, proneurotrophin binding to the p75NTR-sortilin receptor complex is a potent apoptotic stimulus [7, 9, 10] and several studies have demonstrated the importance of proNGF and proBDNF for inducing apoptosis in vivo [7, 10–15]. Further, several groups have reported that proBDNF functions as an important modulator of synaptic structure and function [16, 17].

Although it seems certain that the pro-neurotrophins play physiological roles, the precise signaling pathways that are regulated by these ligands remain uncertain. Recent data have shown that another VPS10 family member, SORCS2, functions as a p75NTR binding partner that acts in a pro-neurotrophin binding complex [18] but the precise signaling cascades and cell biological responses evoked downstream of the p75NTR-sortilin or the p75NTR-SorCS2 complexes remain largely uncharacterized.

Details of proBDNF signaling and cell biology have been slow to emerge, in part due to a lack of robust tools. p75NTR is a member of the TNF receptor superfamily and several major findings on receptors of this class have relied on the development of tagged ligands that can be used to identify and track receptor complexes in cell biological assays [19]. Here, we describe a novel tagged proBDNF isoform that can be easily purified and readily used for tandem affinity purification or for ligand-labeling approaches. This reagent exhibits normal biological activity and can be used to isolate proBDNF receptor complexes. We believe it will prove helpful in the characterization of proBDNF signaling mechanisms.

## Material and Methods

### Animals

All experimental procedures were approved by the McGill University Animal Care Committee and were in compliance with the guidelines of the Canadian Council on Animal Care. Male C57BL/6 mice (6–8 weeks old) and rat Sprague-Dawley (7–8 days old) were obtained from Charles River Canada. Animals were housed under standard conditions with a 12 h light/dark cycle and had free access to water and food.

### Cell culture

HEK293T cells were maintained in Dulbecco's modified Eagle's (DME) medium containing 10% bovine calf serum, 2 mM L-Glutamine and 100 μg/ml penicillin and 100 μg/ml streptomycin in a humidified 37°C incubator with 5% CO_2_. PC12 cells were maintained in DME containing 6% bovine calf serum and 6% deactivated horse serum, 2mM L-glutamine and 100μg/ml penicillin and 100μg/ml streptomycin in a humidified 37°C incubator with 10% CO_2_. Dissociated hippocampal neurons were prepared from embryonic day 15 CD1 mouse embryos (Charles River). Briefly, the hippocampus was dissected out in Hank’s balanced salt solution buffered with Hepes and dissociated via trypsin/EDTA treatment. Cells were seeded on poly-D-lysin coated coverslips and fed Neurobasal media supplemented with 2% B27 (Invitrogen), 1% L-glutamine (Wisent) and 1% penicillin/streptomicyin (Wisent)] and astrocyte conditioned media for 10 days in vitro, prior to treatment with BDNF, proBDNF or HBpFproBDNF. All cell culture reagents were obtained from Hyclone (Logan, UT).

### Treatments and reagents

For phospho-Trk and phospho-Erk activation, CGN and PC12 cells were incubated in serum-free DME supplemented with 0.1% bovine serum albumin (BSA) and 2mM L-glutamine and 100μg/ml penicillin and 100μg/ml streptomycin (DMEB) for 1h prior to treatment with BDNF, proBDNF and HBpF-proBDNF. The anti-biotin antibody was from Jackson Immunoresearch, anti-Flag and anti-beta III-tubulin were from Sigma, anti-BDNF (N-20) was from Santa-Cruz Biotechnology, Inc. and anti phospho-TrkA (Tyr490) was from Cell Signaling Technology. Antibodies against p75NTR, total TrkA and total TrkB have been previously described [20–23]. Anti-Sortilin and anti-SorCS2 were gifts from Dr. Barbara Hempstead, Rhodamine-tagged phalloidin was from Life technologies and Hoechst 33342 was obtained from Molecular Probes. Horseradish-peroxidase-conjugated and fluorescent dye-conjugated secondary antibodies were purchased from Jackson ImmunoResearch (West Grove, PA). BDNF (B250) and proBDNF (B240) was purchased from Alomone labs (Jerusalem, Israel). Cy3-Streptavidin was purchased from Invitrogen and GM6001 was purchased from Calbiochem.

### Generation of recombinant HBpF-proBDNF

Tags were introduced into mouse proBDNF using PCR overlap and the QuikChange Site-Directed Mutagenesis Kit (Agilent). Inserted sequences encoded the following amino acid sequence: 6xHis-tag was HHHHHH; Biotin Acceptor Peptide (BAP) was GLNDIFEAQKIEWH; linker sequence was SGGGGS, PreScission^™^ Protease sequence was LEVLFQG and FLAG was DYKDDDDK. The QuikChange Site-Directed Mutagenesis Kit was used to mutate the furin-cleavage site of proBDNF from KR to AA (nt. 409 to 414). Fidelity of the complete HBpF-proBDNF construct was confirmed by sequencing and the construct was subcloned into pDest12.2 for mammalian expression.

### HBpF-proBDNF production and purification

HEK293T cells were transfected with plasmids encoding HBpF-proBDNF and BirA using calcium phosphate transfection. Media was changed 16 hours later and supplemented with 100μM D-Biotin. 48h later, media was collected and cells were lysed in 20mM Tris (pH 7.5), 300mM NaCl, 10mM imidazole and a protease inhibitor cocktail (Roche). After sonication and centrifugation to remove insoluble components, the supernatant was rotated with Ni-NTA beads (Qiagen) for 1h at 4°C. Beads were washed twice in wash buffer (20mM TRIS pH 7.5, 300mM NaCl, 20mM Imidazole) and then incubated 30 minutes in elution buffer (20mM TRIS pH 7.5, 300mM NaCl, 500mM Imidazole). The eluate was dialysed against 20mM Tris (pH 7.5) + 300mM NaCl and then concentrated in centrifugal filters (Amicon Ultra, Millipore). Protein Stabilizing Cocktail (4x) and Halt Protease Inhibitor Cocktail (100x) (both from ThermoScientific) were added to the concentrated sample and the protein was stored at -80°C. HBpF-proBDNF samples concentration was determined on dot-blots, with commercial BDNF serving as a reference. To produce negative controls, the identical procedure was performed on extracts of HEK293 cells transfected with BirA plasmid alone and final concentrated eluates were used in functional assays. To fluorescently label HBpF-proBDNF, HBpF-proBDNF bound to Ni-NTA beads was incubated with Cy3-Streptavidin (30μg/ml) for 1h at 4°C. After extensive washes, the Cy3-streptavidin: HBpF-proBDNF complex was eluted and concentrated as described above.

### Modified tandem affinity enrichment

PC12 cells were exposed to HBpF-proBDNF, washed, lysed in lysis buffer (20mM Tris (pH7.5), 50mM NaCl, 1mM CaCl_2_, 0.1% Triton-X100 and protease inhibitor cocktail, EDTA-free (Roche)) then rotated with streptavidin beads for 1h at room temperature. After washing twice with lysis buffer, beads were incubated for 16h at 4°C with PreScission^™^ Protease (20mM Tris (pH7.5), 150mM NaCl, 1mM DTT and 0.5mM EDTA), using the manufacturer's protocol (PreScission^™^ Protease, GE). The eluate was then collected, diluted in 4X Laemmli sample buffer, and analyzed by SDS-PAGE.

### Immunolabeling

For immunoblotting, samples were boiled 5 minutes, separated by SDS-PAGE and transferred on nitrocellulose membranes. Membranes were blocked in TBST (10mM TRIS pH8.0, 150mM NaCl, 2% Tween 20) supplemented with 5% dried skim milk powder (or with 2% BSA for immunoblotting phospho-antibodies). Incubation with primary and secondary antibodies was performed in blocking solution. Membranes were washed extensively in TBST after each incubation and immunoreactive bands were detected using enhanced chemiluminescence (ECL, Pierce). For immunocytochemistry, cells were grown on coverslips coated with poly-L-lysine. After treatment, cells were fixed for 30 minutes at room temperature in 4% formaldehyde, permeabilized for 5 minutes with 0.2% Triton X-100 in phosphate buffered saline (PBS), washed once with PBS, blocked for 1h in 0.02% Triton X-100, 1% BSA in PBS and then incubated overnight with the primary antibody diluted in 0.02% Triton X-100, 1% BSA in PBS. The coverslips are washed three times with PBS, then incubated with the secondary antibody in 0.02% Triton X-100, 1% BSA in PBS. Finally coverslip were mounted in an anti-fading mounting media (Dako) and kept at 4°C until imaging.

### Growth cone collapse assay

CGNs were exposed to HBpF-proBDNF for 1 hour then fixed and stained with antibodies directed against beta-III tubulin and phalloidin. Images were collected on a Zeiss AxioCam microscope. Collapsed growth cones are defined as those with no lamellipodia and not more than two filopodia. At least 50 growth cones were analyzed for each data point and statistical significance was established using two-tailed unpaired t-tests.

### Brain slice preparation and electrophysiological recordings

Briefly, 6 to 8 week-old C57BL/6 mice were anaesthetized with ketamine:xylamine cocktail (60 mg/kg) and perfused with ice-cold choline chloride-based artificial cerebrospinal fluid (ACSF) containing (in mM): 110 choline-Cl, 1.25 NaH_2_PO_4_, 25 NaHCO_3_, 7 MgCl_2_, 0.5 CaCl_2_, 2.5 KCl, 7 glucose, 3 pyruvic acid and 1.3 ascorbic acid, pH 7.4 bubbled with carbogen (O_2_ 95%, CO_2_ 5%). Semicoronal horizontal slices (300 μm) containing the entorhinal cortex (EC) from the retrohippocampal region were produced using a VT1000 vibratome (Leica, Ontario, Canada) in the same choline chloride-based solution. Slices were maintained in ACSF containing (in mM): 124 NaCl, 3 KCl, 26 NaHCO_3_, 1.8 MgSO_4_, 1.25 NaH_2_PO_4_, 10 glucose, 1.6 CaCl_2_ for 1h before recording at room temperature (22–24°C) for 1 hour prior to recordings.

For electrophysiology, slices were placed in a recording chamber perfused by gravity at a speed of 1–2 ml/min with ACSF containing kynurenic acid (2 mM) and picrotoxin (100 μM). A near-infrared charged-coupled device camera was used to visualize layer V entorhinal neurons. The temperature of perfusion solution was maintained at 32–34°C. Patch pipettes (5–9 MΩ) were pulled on a Brown Flaming puller (P-97, Sutter Instruments, Novato, CA) using borosilicate glass electrode (Sutter Instruments) and filled with an intracellular solution composed of (in mM): 120 K-gluconate, 10 HEPES, 0.2 EGTA, 20 KCl, 2 MgCl_2_, 7 diTrisP-Creatine, 4 Na_2_ATP and 0.3 NaGTP (pH adjusted to 7.3 with KOH). Tight seals (>5 GΩ) were obtained by applying constant negative pressure. Electrical signals were amplified using an Axopatch 200B amplifier (Molecular Devices, Sunnyvale, CA), low-pass-filtered at 10 kHz, digitized at 50 kHz via a Digitadata 1322A interface (Molecular Devices), and stored on a computer using pClamp9.2 software (Molecular Devices) for off-line analysis. In this study, all neurons displayed a resting membrane potential ranging from −55 to −75 mV. The holding current was around 0 pA and slightly adjusted to obtain a membrane potential of –60 mV. Electrophysiological data were analyzed using Clampfit 9.2.1.8 (Axon instruments). Values are expressed as mean +/- S.E.M. Firing frequency was defined as the average spiking frequency within 20 s after the depolarizing current pulse (1 s duration, 100 pA). Plateau amplitude was defined as the difference between the mean membrane potential (mV) measured at baseline before the pulse and the mean membrane potential measured during the steady-state phase of persistent firing.

## Results

### Construction, production, and purification of HBpF-proBDNF

Tandem affinity purification (TAP) has emerged as a powerful technique for identifying protein-protein interactions and has been widely used as method for identifying protein complexes. The basic method involves placing two tags on a protein, typically separated by a proteolytic site; affinity purification is performed via the distal tag, the bound protein is then released from its support using a protease that targets the cleavage site between the tags, and the complex is further enriched by affinity purification against the remaining tag [24, 25].

**Fig 1:**
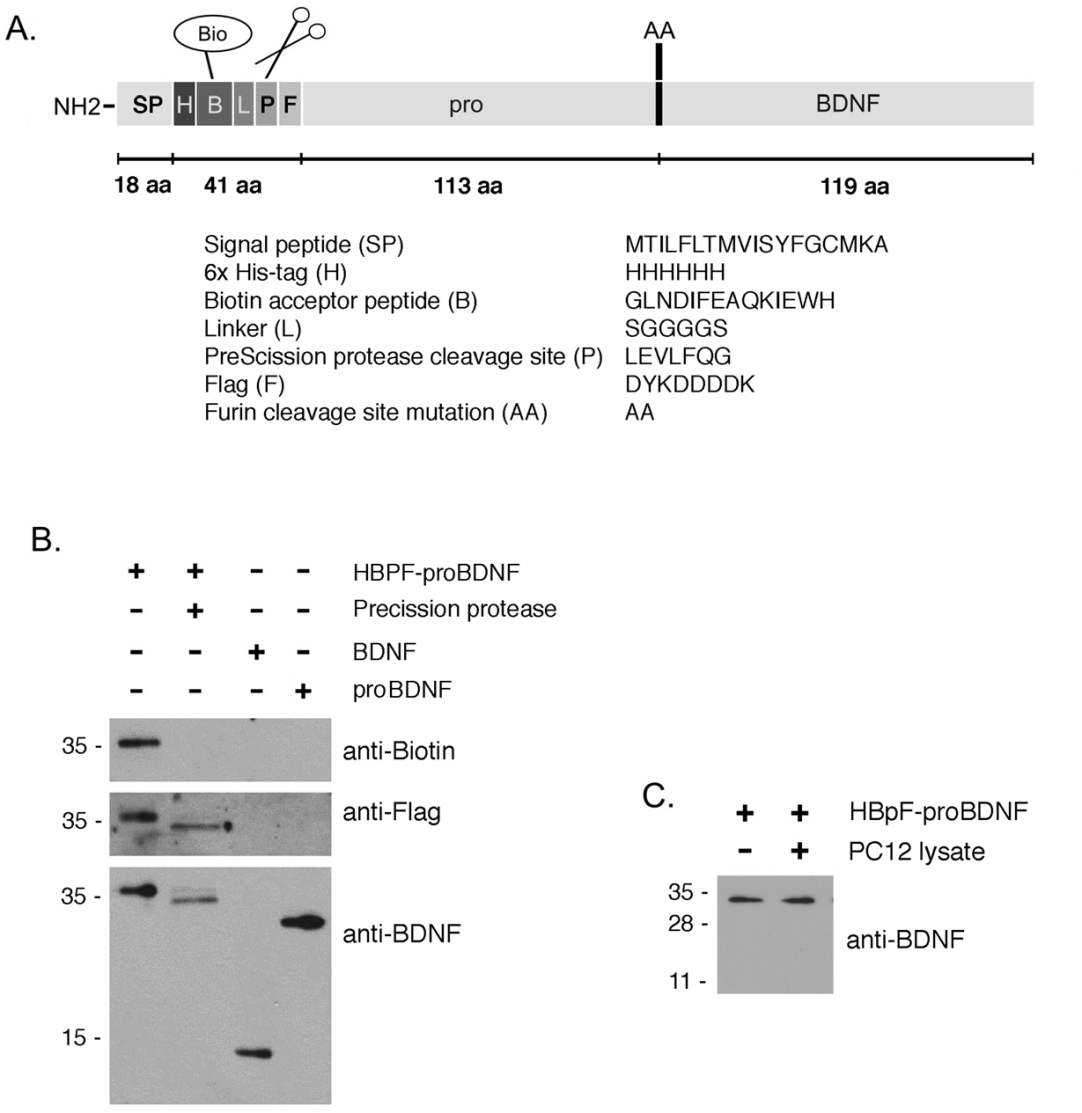
HBpF-proBDNF design and production. A. Schematic representation of recombinant HBpF-proBDNF protein. HBpF-proBDNF contains a signal peptide, an amino-terminal 6xHis-tag, followed by a Biotin-Acceptor-Peptide (BAP) sequence, a linker (L), a PreScission^™^ Protease (PP) cleavage site and a Flag-tag (Flag). The ProBDNF sequence has been mutated with a KR to AA mutation at the furin dibasic cleavage site between the pro-domain and the mature part of BDNF. B. HEK293T cells were transfected with HBpF-proBDNF and BirA plasmids. After Ni-NTA pulldown and cleavage with the PreScission^™^ Protease, the eluates were analyzed by Western blot and blotted with anti-biotin, anti-Flag and anti-BDNF. C. Purified HBpF-proBDNF was incubated with PC12 cells lysates, without a protease inhibitors, for 1h at room temperature. Incubation of HBpF-proBDNF with lysis buffer for 1h at room temperature was used as a control. Immunoblots against BDNF were performed to visualize the degradation of HBpF-proBDNF.

Here, we developed a TAP-compatible form of proBDNF that has several unique features (Fig 1A). From the N-terminal end, HBpF-proBDNF consists of the endogenous gene’s signal peptide, a 6xHis-tag (H), a biotin acceptor peptide (B), a linker, a PreScission^™^ Protease cleavage site (p) and a Flag-tag (F); this extension is followed by murine proBDNF containing two site directed mutations, R129A and R130A. The 6xHis-tag is used to purify HBpF-proBDNF from conditioned media or from mammalian cell lysates with Ni-NTA beads (Fig 1A). The biotin acceptor peptide allows HBpF-proBDNF to be metabolically biotinylated in cells expressing a BirA biotin ligase and enables high-stringency affinity purification on streptavidin or can be used to label ligand with avidin-tagged fluorophores. Bound HBpF-proBDNF and associated proteins are released from streptavidin beads by cleavage by PreScission^™^ Protease at 4°C. This cleavage allows Flag-tagged proBDNF (F-proBDNF) to be generated and allows it and associated binding partners to be recovered as an eluted product with a high degree of purification. The R129A/R130A mutations remove the native dibasic site that is cleaved to produce BDNF from proBDNF.

HBpF-proBDNF was overexpressed in HEK293T cells together with BirA biotin ligase in media supplemented with 100μM biotin. After 48 hours, cells were lysed and incubated with Ni-NTA beads for 2 hours and washed extensively. Bound HBpF-proBDNF was then eluted with 500mM imidazole or exposed to PreScission^™^ Protease (1 unit/100μg of protein) for 12 hours at 4°C. The primary HBpF-proBDNF product is mainly a 35kDa protein, with a less abundant subsidiary product of 33 kDa. We have not directly assessed HBpF-proBDNF glycosylation but based on a previous analysis, it is likely that two isoforms reflect distinct proBDNF glycosylation states [26]. Both isoforms are readily detected by anti-biotin, anti-FLAG and anti-BDNF antibodies (Fig 1B, lane 1). After exposure to precision protease, the predominant and subsidiary product shift to molecular weights of 32 kDa and 30 kDa, respectively. As expected, these products are detected by anti-FLAG and anti-BDNF but not by anti-biotin and correspond to liberated Flag-proBDNF isoforms (F-proBDNF). To test if proBDNF can become cleaved during the process of cell lysis, HBpF-proBDNF bound to Ni-NTA beads was exposed to cell lysates, without protease inhibitor for 1 hour at room temperature. Fig 1C shows that HBpF-proBDNF remained intact under these conditions.

### Biological properties of HBpF-proBDNF

**Fig 2:**
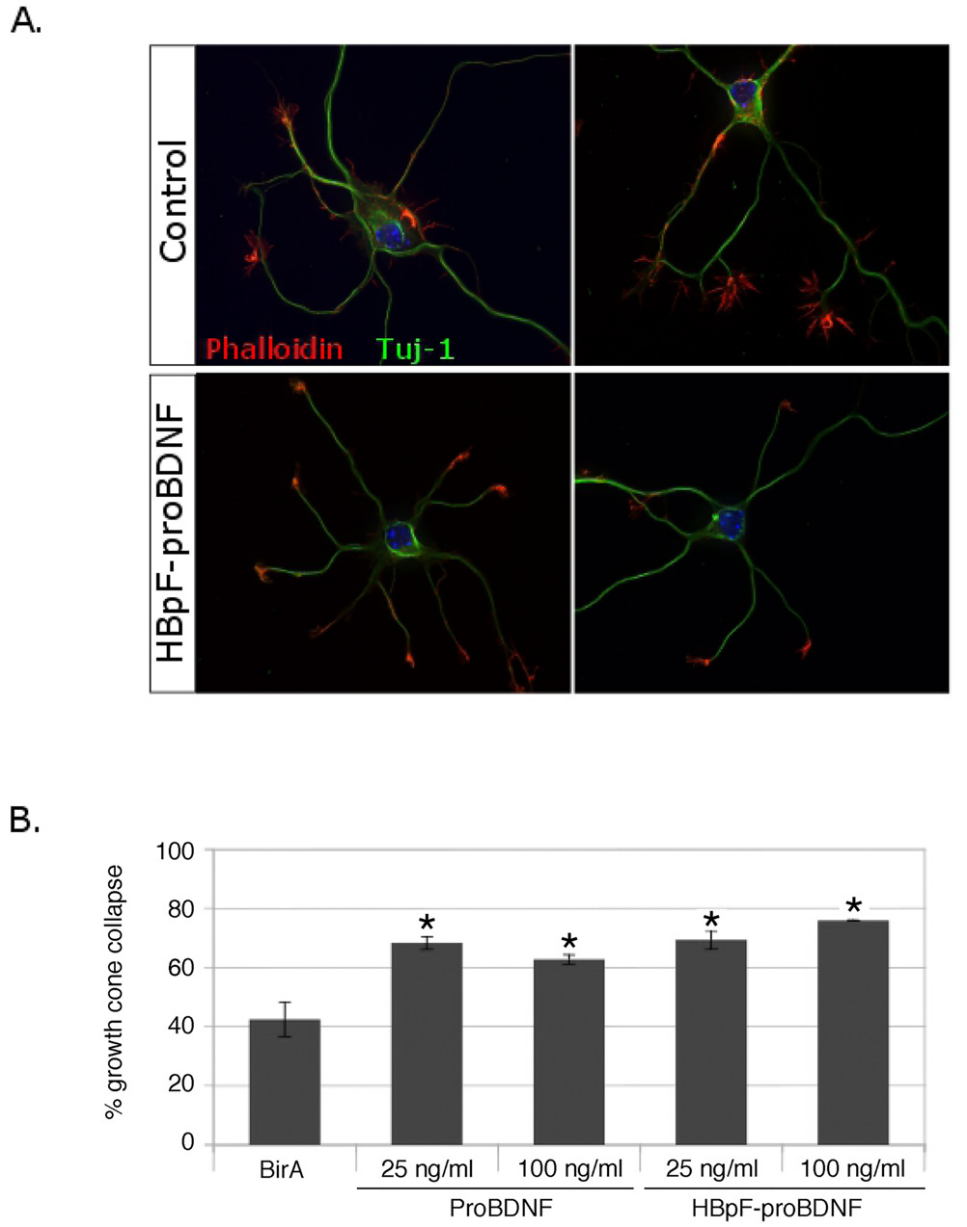
HBpF-proBDNF induces growth cone collapse. Following 2 days in culture, hippocampal neuronal culture were stimulated with different concentrations of HBpF-proBDNF or proBDNF (25ng/ml and 100ng/ml) for 1h. Ni-NTA eluate from cells expressing only BirA was used as a negative control. Cells were then fixed and immunostained against beta-III-tubulin (Tuj-1) and phalloidin (scale bar = 10μm). Quantification of growth cone-collapse was done on three independent experiments and 50 growth-cones were counted for each experiment (unpaired two-tailed *t*-test, * indicates a p-value < 0.05; bars indicate standard error).

Recent studies have indicated that proBDNF induces growth cone collapse in murine dorsal root ganglion (DRG) neurons and in cortical neurons [15]. To determine if purified recombinant HBpF-proBDNF can elicit this effect in central neurons, we evaluated growth cone collapse in hippocampal neurons. Hippocampal neurons were left untreated or were exposed to purified HBpF-proBDNF (25ng/ml and 100ng/ml) for one hour. As a positive control, we tested identical concentrations of commercial proBDNF (Alomone Labs) and as a negative control, we exposed cells to eluates derived from cells expressing only BirA (see Methods). Quantification of growth cone collapse showed that the BirA eluate had no effect on growth cone morphology whereas both HBpF-proBDNF and commercial proBDNF increased hippocampal growth cone collapse (Fig 2A and 2B). The dose required to elicit the effect was similar with the two preparations, with 25 ng/ml increasing growth cone collapse by ~40%.

**Fig 3:**
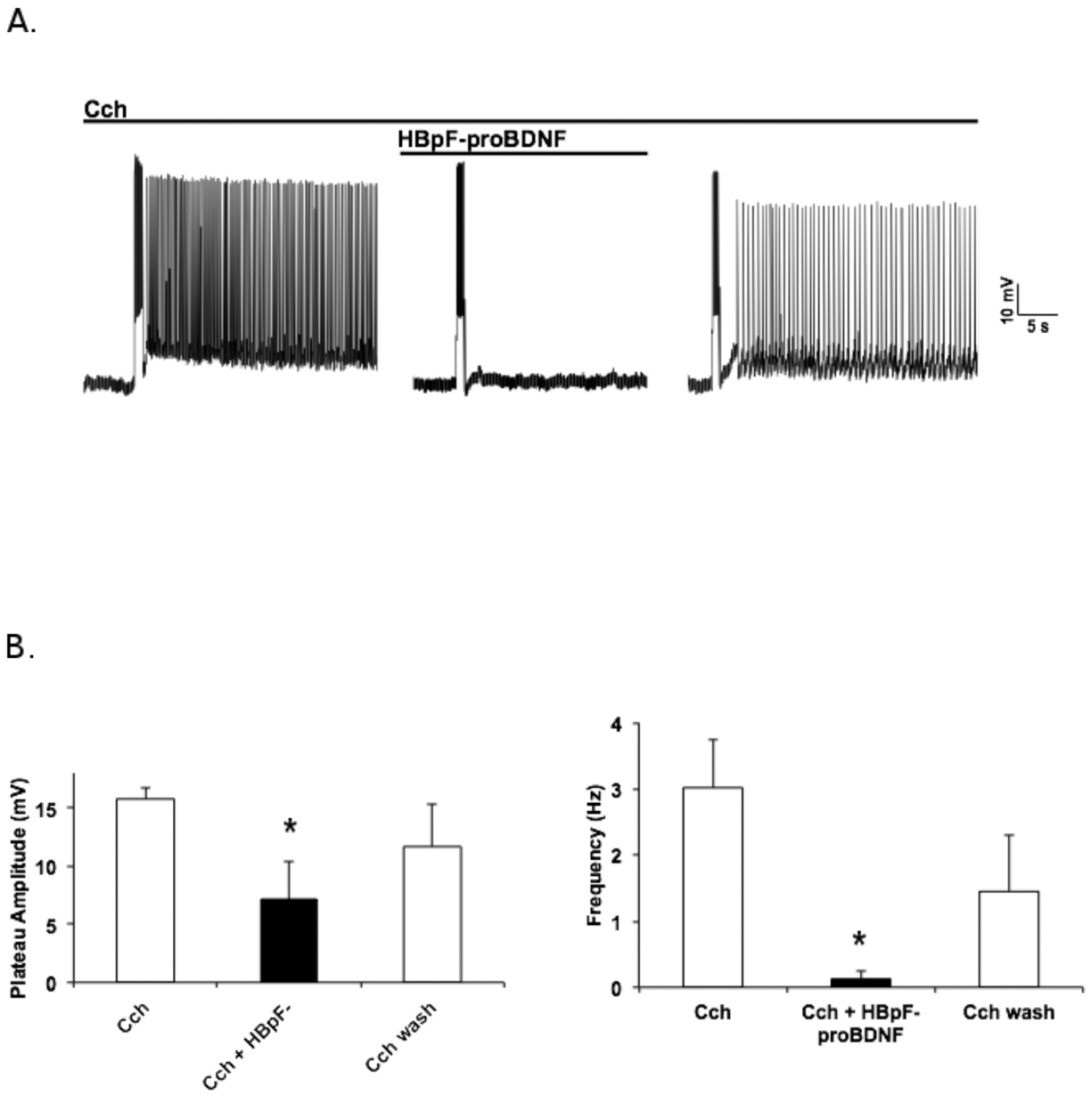
HBpF-proBDNF inhibits carbachol (CCh)-induced persistent firing in cortical pyramidal neurons. A. Representative trace of current-clamp recording from pyramidal neuron in layer V of the entorhinal cortex. Slices were perfused with 10μM CCh and the persistent activity was produced by a short depolarization (1s, 100pA). HBpF-proBDNF at 2ng/ml was next added in presence of 10μM CCh during 10 minutes (first cut in the trace) and cells were stimulated. HBpF-proBDNF was removed by perfusing a solution containing only 10μM CCh for 10 minutes (second cut in the trace) and before the stimulation of the cells. B. Quantification of the plateau amplitude and frequency of the persistent activity (unpaired two-tailed *t*-test, * indicates a p-value < 0.05; bars indicate standard error).

We have recently shown that proBDNF, working through p75NTR, has a profound effect on the excitability of layer V pyramidal neurons in the entorhinal cortex [27]. These neurons normally exhibit persistent firing when exposed to the muscarinic agonist carbachol but this effect is strongly suppressed in the presence of commercial proBDNF (Fig 3A). To test if HBpF-proBDNF is also capable of suppressing persistent activity, murine entorhinal cortical slices were perfused with 10μM carbachol (CCh) for 10 minutes, stimulated with 100pA of depolarizing current for 1s and recordings were performed in whole cell patch clamp. The same slices were then exposed to HBpF-proBDNF (2ng/ml). Fig 3A shows that the persistent activity induced in slices incubated in 10μM carbachol is strongly suppressed by HBpF-proBDNF, with both plateau amplitude and firing frequency significantly decreased upon HBpF-proBDNF exposure (Fig 3B). Importantly, this effect is reversible as subsequent washout of HBpF-proBDNF restores persistent activity. Thus, HBpF-proBDNF is functionally indistinguishable from wild-type proBDNF in this setting.

**Fig 4:**
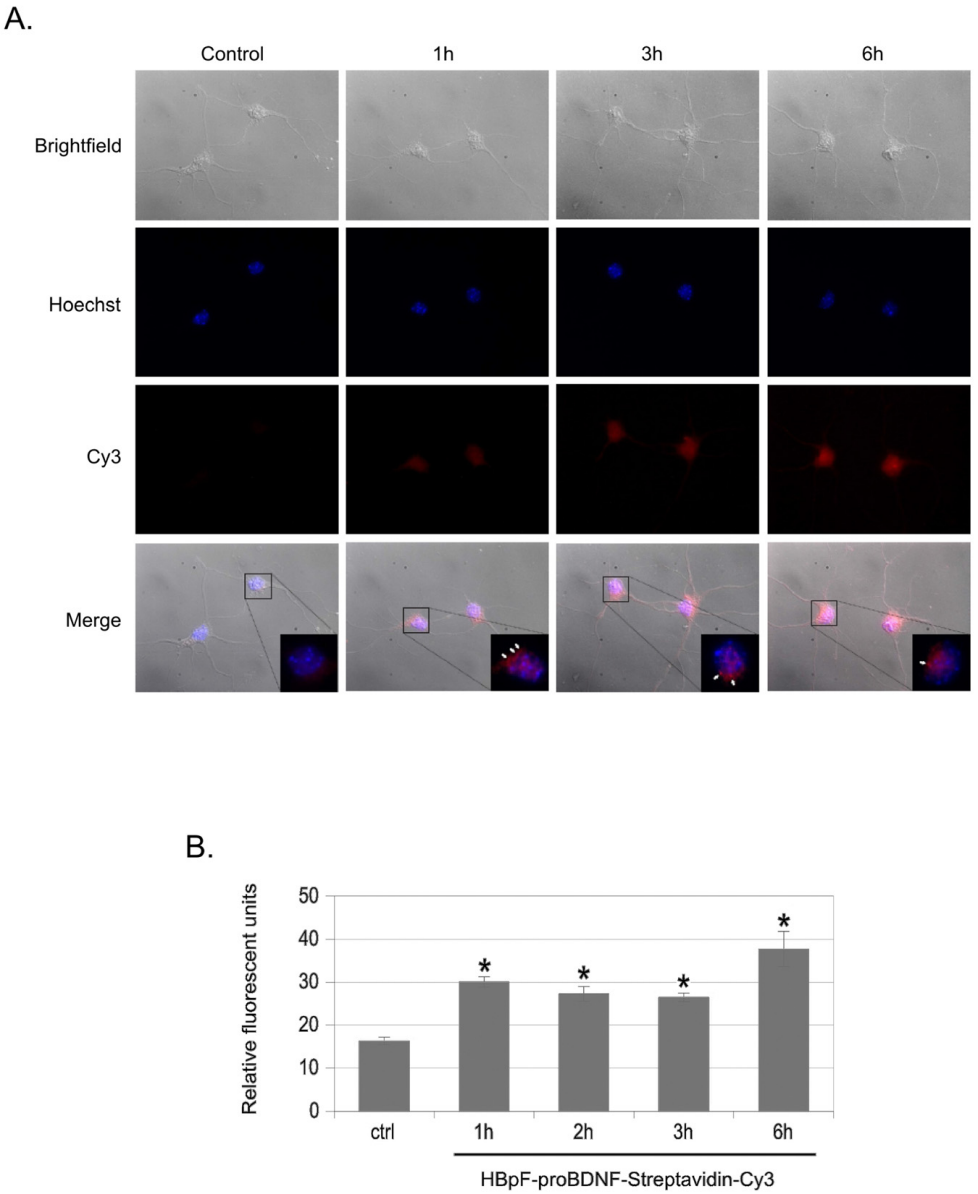
Endocytosis of HBpF-proBDNF in Hippocampal neurons. Primary hippocampal neurons cells were exposed to HBpF-proBDNF (250 ng/ml) conjugated to Streptavidin-Cy3 for 1h to 6h. After fixation and mounting, cells were analyzed by fluorescent microscopy. Hoechst staining was used as a nuclear marker. Quantification of fluorescence intensity was performed using ImageJ software on three independent experiments. 100 cells were measured for each experiment. (unpaired two-tailed *t*-test, * indicates a p-value < 0.05; bars indicate standard error).

Previous studies have established that proBDNF bound to fluorescently labeled antibodies is endocytosed through a p75NTR-dependent pathway [9]. To determine if HBpF-proBDNF could also be endocytosed, we first labelled HBAP-proBDNF with the fluorophore Cy3-Streptavidin. For this, biotinylated HBpF-proBDNF was bound to a Ni-NTA resin and then incubated with a Cy3-Streptavidin conjugate for 1 hour, washed extensively, and then eluted with imidazole. After dialysis and quantification, Cy3-labelled HBpF-proBDNF was added to primary cultures of hippocampal neurons for periods ranging from 1–6 hours. At the end of the incubation period, cells were washed in low pH buffer to remove surface ligand and then quantified by fluorescence microscopy. Cy3-labelled HBpF-proBDNF is clearly internalized after 1 hour of incubation and levels remain stably elevated at later incubation times; unconjugated Cy3-streptavidin, the negative control for these experiments, did not accumulate in hippocampal neuronal cells at any timepoint examined (Fig 4 and data not shown).

**Fig 5:**
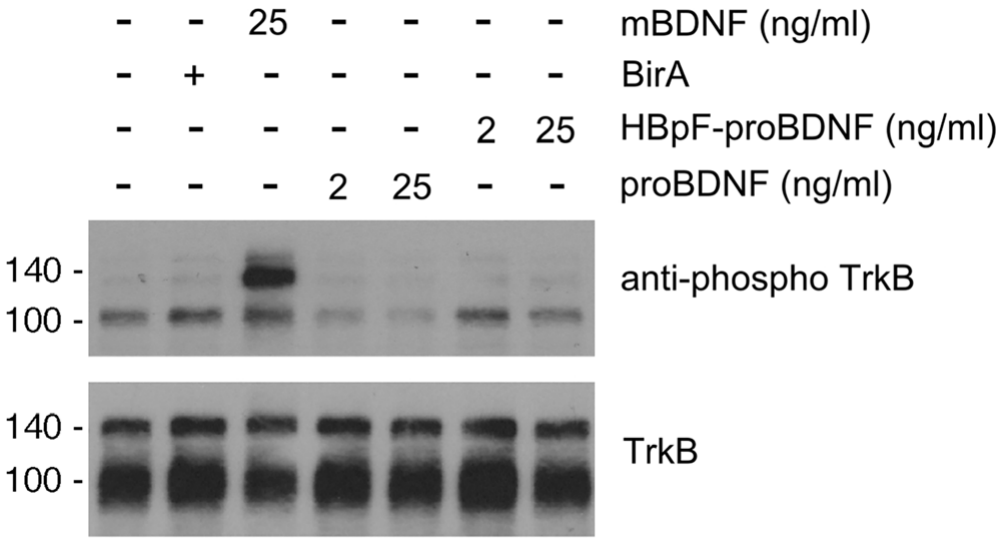
HBpF-proBDNF does not activate TrkB receptors. A. Following 2 days in culture, cerebellar granule neurons were stimulated with different concentrations of HBpF-proBDNF (2ng/ml; 25ng/ml and 100ng/ml) or proBDNF (2ng/ml and 25ng/ml) for 30min. After the incubation time, cells were lysed immediately in sample buffer and analyzed by Western blot against phospho-Trk and TrkB. For positive and negative controls, CGN were treated with BDNF (25ng/ml) or with BirA Ni-NTA eluate (BirA), as indicated.

Mature-BDNF (mBDNF) binds TrkB and p75NTR whereas proBDNF selectively binds a complex containing p75NTR and sortilin or p75NTR and SorCS2 [2, 3, 28, 29]. To confirm that HBpF-proBDNF does not activate TrkB, we exposed CGNs to mBDNF (25ng/ml), to commercial proBDNF (2 and 25ng/ml), and to HBpF-proBDNF (2 and 25ng/ml) for 30 min and then performed immunoblots for phospho-TrkB and total TrkB. Fig 5A shows that mBDNF induces robust TrkB phosphorylation whereas commercial proBDNF and HBpF-proBDNF had no effect on TrkB phosphorylation.

### HBpF-proBDNF can be used to isolate p75NTR complexes

**Fig 6:**
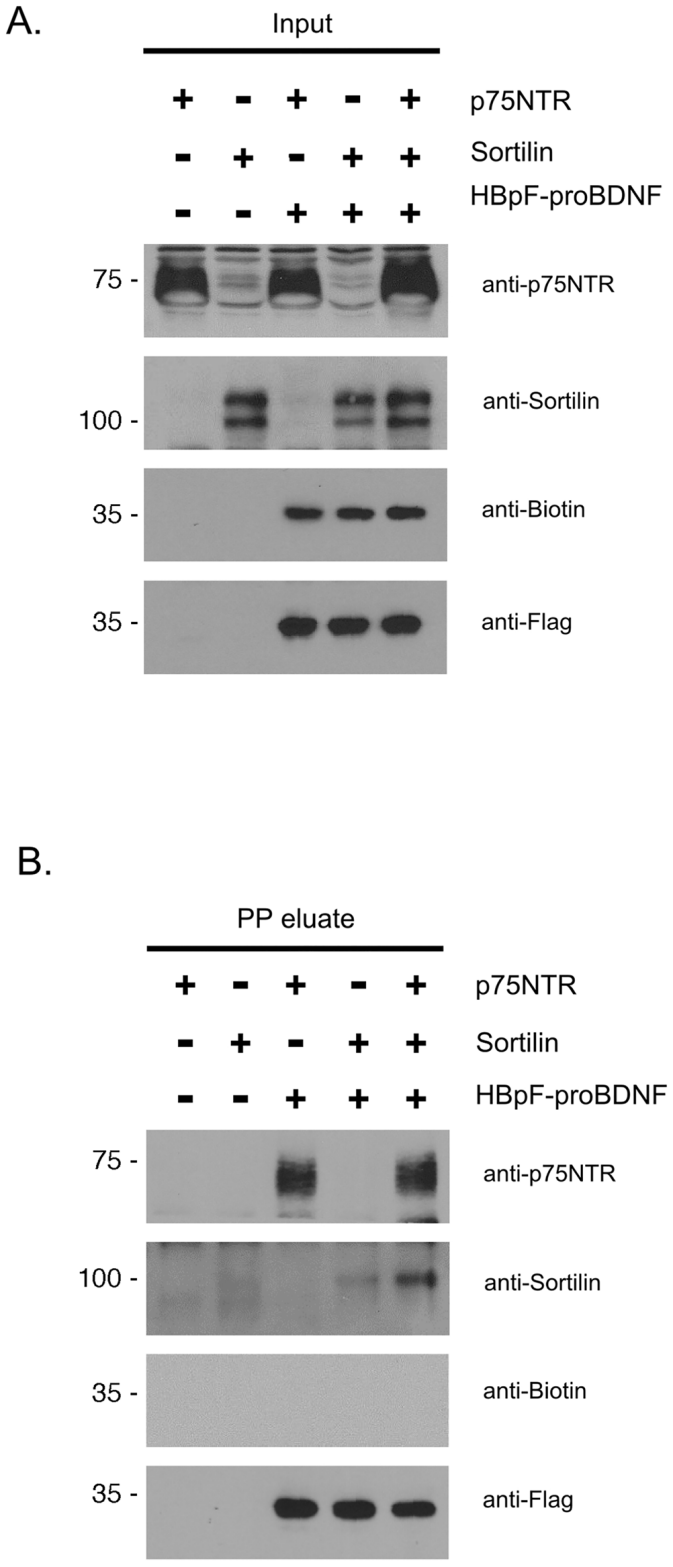
HBpF-proBDNF can be isolated by a modified tandem affinity purification protocol. A. HEK293T cells were transfected with p75NTR, Sortilin, HBpF-proBDNF, and BirA expression plasmid as indicated. 48h after transfection, HEK293T cells were lysed (input) and pulled-down on Ni-NTA beads. The Ni-NTA eluate was then pulled-down on SA beads and then cleaved by PP overnight (PP eluate). Samples were analyzed by immunoblotting for p75NTR, sortilin, biotin and the Flag tag.

HBpF-proBDNF was designed to identify signaling complexes that are formed by proBDNF binding to p75NTR. To confirm that HBpF-proBDNF binds to p75NTR, we overexpressed p75NTR and sortilin either alone or together in HEK293 cells, with and without HBpF-proBDNF. Cells were lysed 48h after transfection, HBpF-proBDNF was purified on Ni-NTA beads, eluted using imidazole, bound to streptavidin beads, then incubated with PreScission^™^ Protease. Proteins released from the beads by PreScission^™^ Protease cleavage (PP eluate) were then collected and analyzed by immunoblot. Fig 6A shows that HBpF-proBDNF was detected by biotin and Flag antibodies whereas HBpF-proBDNF present in the precision protease eluate was detected by anti-Flag but not by anti-biotin antibodies (Fig 6B). This demonstrates that the protease treatment effectively cleaved the biotinylated moiety from HBpF-proBDNF to release F-proBDNF from the beads. Interestingly, p75NTR is co-purified with F-proBDNF in cells lacking or expressing sortilin whereas sortilin levels present within the F-proBDNF eluate were considerably higher in the presence of p75NTR. Thus, the sortilin-proBDNF complex appears to be stabilized in the presence of p75NTR.

**Fig 7:**
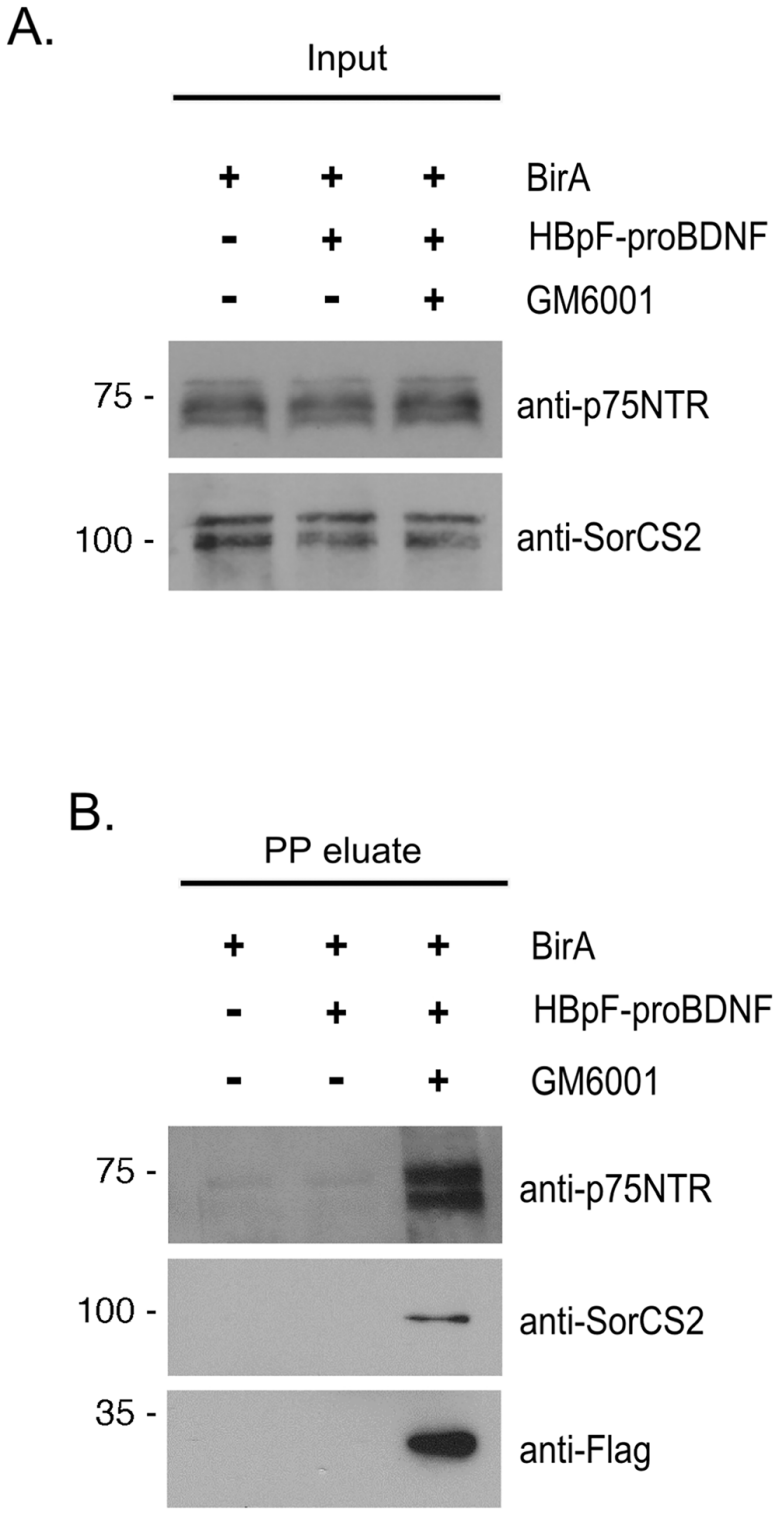
HBpF-proBDNF interacts with endogenous p75NTR and SorCS2 in PC12 cells. PC12 cells were stimulated with HBpF-proBDNF (100μg/ml) with or without 9μM GM6001 for 3h. BirA Ni-NTA eluate was used as control treatment. After stimulation, cells were lysed and HBpF-proBDNF and associated protein were recovered on SA beads. Cleavage with PP was performed for 16 hours and the resulting PP eluate was collected. Cell lysates (Input) and PP eluate samples were then analyzed by immunoblotting for p75NTR, SorCS2 and FLAG.

To determine if HBpF-proBDNF could be used to isolate endogenous receptor complexes in untransfected cells, PC12 cells were exposed to HBpF-proBDNF for 3 hours, then lysed and subjected to streptavidin pulldown followed by cleavage with PreScission^™^ Protease. Since metalloproteases can cleave proBDNF or p75NTR, in parallel we tested whether adding GM6001, an inhibitor of metalloproteases increased the yield of endogenous p75NTR or SorCS2 pulled out using this procedure. Fig 7 shows that p75NTR and SorCS2 were readily co-purified in a complex with HBpF-proBDNF but only in cells incubated with GM6001 (9μM). Interestingly, HBpF-proBDNF was not detected in pullouts from cells lacking GM6001, indicating that endogenous metalloproteases may target bound HBpF-proBDNF for destruction (Fig 7).

## Discussion

ProBDNF has emerged as regulator of cell morphology, neuronal excitability and cell death [2, 30]However, knowledge of the signaling mechanisms activated by this peptide remains rudimentary. Here, we describe the development and characterization of a multi-tagged form of proBDNF that can be used in cell biological assays and in for biochemical experiments that are designed to identify and characterize receptor complexes.

We show that HBpF-proBDNF can be readily over-expressed in HEK293 cells and easily affinity purified by a Ni-NTA procedure. Furthermore, the intact tagged HBpF-proBDNF construct functions identically to its untagged counterpart within *in vitro* settings. Pro-BDNF has been shown to induce growth-cone collapse [5, 13, 31, 32] and we found that HBpF-proBDNF elicits the same response. We have recently shown that activation of the proBDNF -p75NTR transduction cascade inhibits persistent firing in the entorhinal cortex [27] and here, we demonstrated that HBpF-proBDNF reproduces the electrophysiological effect of proBDNF in this setting. Moreover HBpF-proBDNF bound to Cy3-streptavidin was readily endocytosed by hippocampal neuronal cells and did not induce TrkB activation in CGNs. Thus, HBpF-proBDNF exhibits the receptor activation profile previously established for proBDNF.

The effects of proBDNF reported here occur over a large concentration range, consistent with previous studies. In the growth cone collapse assays performed by Sun et al (2012), proBDNF elicited maximal effects at 30 and 100 ng/ml [15]similar to the 25 and 100 ng/ml concentrations used here. We recently examined the effect of proBDNF on persistent activity in entorhinal cortical neurons and in this cell type, maximal effects were elicited at considerably lower proBDNF concentrations (2 ng/ml) [27]. We speculate tat cell-specific differences in proBDNF dose-dependency may reflect the existence of unique p75NTR-containing proBDNF receptor complexes in various cell types.

We also show that HBpF-proBDNF can be used to identify receptor complexes. Using a two-step purification procedure (streptavidin pulldown of the biotinylated HBpF-proBDNF then cleavage of the construct by the PreScission^™^ Protease), we established that HBpF-proBDNF can bind to a complex of p75NTR and sortilin when they are overexpressed in HEK293 cells and it interacts with endogenous p75NTR and SorCS2 in PC12 cells.

Epitope tagged ligands have proven to be valuable tools for analyzing receptor complexes, especially in the TNF receptor superfamily field. For example, early studies employed ligands tagged with a single FLAG or Fc tag [24, 25] showed that TNF receptor I-mediated apoptosis occurs through activation of two sequential signaling complexes. More recent studies, using tagging procedures similar to that described here, resulted in the identification of LUBAC, a receptor-associated linear ubiquitin chain assembly complex [19, 33]. Hence HBpF-proBDNF will be a useful reagent for identifying proBDNF receptor complexes and for unraveling specific signaling mechanisms that mediate the various physiological functions of proBDNF.

## Abbreviations

CGNs: Cerebellum Granular Neurons
BAP: Biotin Acceptor Peptide
PP: PreScission^™^ Protease
NGF: nerve growth factor
BDNF: brain-derived neurotrophic factor
